# Is the generalizability of a developed artificial intelligence algorithm for COVID-19 on chest CT sufficient for clinical use? Results from the International Consortium for COVID-19 Imaging AI (ICOVAI)

**DOI:** 10.1007/s00330-022-09303-3

**Published:** 2023-01-18

**Authors:** Laurens Topff, Kevin B. W. Groot Lipman, Frederic Guffens, Rianne Wittenberg, Annemarieke Bartels-Rutten, Gerben van Veenendaal, Mirco Hess, Kay Lamerigts, Joris Wakkie, Erik Ranschaert, Stefano Trebeschi, Jacob J. Visser, Regina G. H. Beets-Tan, On behalf of the ICOVAI, International Consortium for COVID-19 Imaging AI, Julien Guiot, Annemiek Snoeckx, Peter Kint, Lieven Van Hoe, Carlo Cosimo Quattrocchi, Dennis Dieckens, Samir Lounis, Eric Schulze, Arnout Eric-bart Sjer, Niels van Vucht, Jeroen A.W. Tielbeek, Frank Raat, Daniël Eijspaart, Ausami Abbas

**Affiliations:** 1 Department of Radiology, The Netherlands Cancer Institute, Plesmanlaan 121, 1066 CX Amsterdam, The Netherlands; 2 GROW School for Oncology and Reproduction, Maastricht University, Universiteitssingel 40, 6229 ER Maastricht, The Netherlands; 3 Department of Thoracic Oncology, The Netherlands Cancer Institute, Plesmanlaan 121, 1066 CX Amsterdam, The Netherlands; 4 Department of Radiology, University Hospitals Leuven, Herestraat 49, 3000 Leuven, Belgium; 5 Aidence, Amsterdam, The Netherlands; 6 Department of Radiology, St. Nikolaus Hospital, Hufengasse 4-8, 4700, Eupen, Belgium; 7 Ghent University, C. Heymanslaan 10, 9000 Ghent, Belgium; 8 Department of Radiology and Nuclear Medicine, Erasmus MC, University Medical Center Rotterdam, Dr. Molewaterplein 40, 3015 GD Rotterdam, The Netherlands; 9 Institute of Regional Health Research, University of Southern Denmark, Campusvej 55, 5230 Odense, Denmark

**Keywords:** Artificial intelligence, COVID-19, Computed tomography, Reproducibility of results, Validation study

## Abstract

**Objectives:**

Only few published artificial intelligence (AI) studies for COVID-19 imaging have been externally validated. Assessing the generalizability of developed models is essential, especially when considering clinical implementation. We report the development of the International Consortium for COVID-19 Imaging AI (ICOVAI) model and perform independent external validation.

**Methods:**

The ICOVAI model was developed using multicenter data (*n* = 1286 CT scans) to quantify disease extent and assess COVID-19 likelihood using the COVID-19 Reporting and Data System (CO-RADS). A ResUNet model was modified to automatically delineate lung contours and infectious lung opacities on CT scans, after which a random forest predicted the CO-RADS score. After internal testing, the model was externally validated on a multicenter dataset (*n* = 400) by independent researchers. CO-RADS classification performance was calculated using linearly weighted Cohen’s kappa and segmentation performance using Dice Similarity Coefficient (DSC).

**Results:**

Regarding internal versus external testing, segmentation performance of lung contours was equally excellent (DSC = 0.97 vs. DSC = 0.97, *p* = 0.97). Lung opacities segmentation performance was adequate internally (DSC = 0.76), but significantly worse on external validation (DSC = 0.59, *p* < 0.0001). For CO-RADS classification, agreement with radiologists on the internal set was substantial (kappa = 0.78), but significantly lower on the external set (kappa = 0.62, *p* < 0.0001).

**Conclusion:**

In this multicenter study, a model developed for CO-RADS score prediction and quantification of COVID-19 disease extent was found to have a significant reduction in performance on independent external validation versus internal testing. The limited reproducibility of the model restricted its potential for clinical use. The study demonstrates the importance of independent external validation of AI models.

**Key Points:**

*• The ICOVAI model for prediction of CO-RADS and quantification of disease extent on chest CT of COVID-19 patients was developed using a large sample of multicenter data.*

*• There was substantial performance on internal testing; however, performance was significantly reduced on external validation, performed by independent researchers. The limited generalizability of the model restricts its potential for clinical use.*

*• Results of AI models for COVID-19 imaging on internal tests may not generalize well to external data, demonstrating the importance of independent external validation.*

**Supplementary Information:**

The online version contains supplementary material available at 10.1007/s00330-022-09303-3.

## Introduction

Artificial intelligence (AI)-based analysis of imaging performed for coronavirus disease 2019 (COVID-19) evaluation has been extensively researched [1]. During the pandemic, several deep learning models have been developed, aiming to assist radiologists in interpreting and reporting chest CT scans in COVID-19 patients.

Volume quantification of affected lung tissue on chest CT scans has been shown to correlate with disease severity in COVID-19 [2–6]. Manual delineation of lung abnormalities by radiologists is labor-intensive and time-consuming, and therefore not routinely conducted in clinical practice. Automated segmentation of affected lung tissue can be made readily available, thereby allowing clinical adoption of quantitative analysis.

To standardize reporting of chest CT scans, the COVID-19 Reporting and Data System (CO-RADS) was introduced [7]. The grading system includes five categories of increasing disease probability, ranging from negative (CO-RADS 1) to typical imaging findings of COVID-19 (CO-RADS 5). CO-RADS has shown reasonable to very good diagnostic performance and interobserver agreement [7–10]. Applying machine learning techniques to automate CO-RADS classification could potentially improve the interobserver agreement, especially for less experienced readers. Moreover, such an automated analysis can be performed before clinicians have the opportunity to read the CT scan, ensuring the CO-RADS classification and volume quantification are present at the time of interpretation. This could potentially result in a more efficient clinical workflow if the automated assessment is sufficiently accurate.

Before any AI application is considered for widespread clinical use, external validation of the model should be performed [11]. In the systematic review by Roberts et al, only 8 of 37 (22%) deep learning papers on COVID-19 imaging analysis that passed their quality check had completed external validation [12]. This might especially be worrisome for AI applications in COVID-19 imaging since several methodological flaws and biases in these studies were reported [12]. The authors stressed the importance of performing an external validation on a well-curated dataset of appropriate size to evaluate the generalizability of an AI model, ensuring it translates well to unseen, independent data.

This study aimed to develop and independently validate an AI model consisting of COVID-19 segmentation and likelihood estimation (CO-RADS) on chest CT using multicenter data.

## Material and methods

### International Consortium for COVID-19 Imaging AI (ICOVAI)

During the initial phase of the COVID-19 pandemic, there was a need for accurate and efficient analysis of chest CT scans. ICOVAI was formed to address this need. The collaboration consisted of multiple hospitals and industry partners across Europe. The consortium aimed to develop an AI-based quantification and CO-RADS classification tool for clinical use, following good-practice guidelines. These principles included high-quality diverse data and multiple expert readers to perform data annotation.

### Data collection

**Fig. 1:**
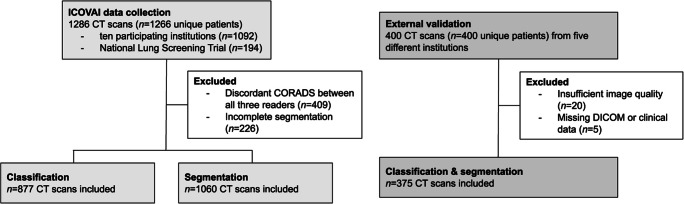
Data flowchart for the ICOVAI model development and external validation

The ICOVAI consortium included a multicenter, international cohort of patients suspected of COVID-19 pneumonia undergoing chest CT. The dataset for model creation consisted of *n* = 1092 CT scans of patients with available reverse transcriptase-polymerase chain reaction (RT-PCR) test results for COVID-19 (*n* = 580 positive, *n* = 512 negative), shown in Fig. 1. The data was collected between December 2019 and May 2020 through ten participating institutions (Table 1). The male (*n* = 545) to female (*n* = 547) ratio was 1:1. To balance the dataset, *n* = 194 CT scans from the National Lung Screening Trial were added as negative control samples. Combined, the total dataset yielded *n* = 1286 CT scans from *n* = 1266 unique patients.

**Table 1:** Dataset of the ICOVAI consortium. Number of CT scans per participating institution for both the classification and segmentation task. The data is split into a training and internal test set for both tasks

Institution	Classification		Segmentation	
	Training	Internal test	Training	Internal test
Albert Schweitzer Hospital, The Netherlands	15	1	20	1
AZ Turnhout, Belgium	82	4	102	7
Catharina Hospital, The Netherlands	19	3	26	3
Imapôle Lyon-Villeurbanne, France	108	17	136	19
Laurentius Hospital, The Netherlands	145	9	179	17
Lifetrack, Singapore	1	0	2	0
NHSX, UK	50	7	59	9
Rijnstate, The Netherlands	14	3	22	3
Tergooi MC, The Netherlands	13	0	13	0
Franciscus Gasthuis & Vlietland, The Netherlands	216	14	252	16

*ICOVAI* International Consortium for COVID-19 Imaging AI

An independent test dataset for external validation was retrospectively collected from five different hospitals in Europe (Table 2). The cohort included *n* = 400 adult patients undergoing chest CT for suspected COVID-19 pneumonia or triage between February 2020 and May 2020. Twenty-five patients were excluded due to severe breathing or motion artifacts (*n* = 9), insufficient inspiration (*n* = 9), low resolution (*n* = 2), or missing DICOM data or clinical information (*n* = 5). After exclusion, *n* = 375 CT scans of unique patients remained, with a mean age of 61.1 years (SD 16.8), and male-to-female ratio of 1.1:1. The majority of patients showed symptoms of respiratory infection at the time of imaging (*n* = 332, 88.5%). RT-PCR tests performed within 7 days of imaging were used as a reference standard and available for *n* = 363 patients (96.8%). Available RT-PCR test results were positive for *n* = 181 patients and negative for *n* = 182 patients.

**Table 2:** Dataset for external validation. Number of CT scans per participating institution

Institution	External test
Amphia Hospital, The Netherlands	56
Antwerp University Hospital, Belgium	171
Campus Bio-Medico University of Rome, Italy	15
University Hospital of Liège, Belgium	87
OLV Hospital, Belgium	46
Total	375

### Data annotation

ICOVAI model: Multiple radiologists independently classified all CT scans (*n* = 1286) using the CO-RADS scheme (*n* = 1058 by three readers, *n* = 228 by two readers). A total of 409 cases were excluded due to discordance, i.e., all readers yielded different CO-RADS scores, resulting in 877 CT scans. The distribution of classification labels for both the training (*n* = 805) and internal test set (*n* = 72) is shown in Table 3.

**Table 3:** Distribution of CO-RADS categories per dataset

CO-RADS	Training	Internal test	External test
1	362 (45%)	30 (42%)	137 (37%)
2	121 (15%)	12 (17%)	69 (18%)
3	66 (8%)	6 (8%)	48 (13%)
4	60 (7%)	6 (8%)	13 (3%)
5	196 (24%)	18 (25%)	108 (29%)
Total	805	72	375

*CO-RADS* COVID-19 Reporting and Data System

The total lung volume and lung opacities were manually segmented by medical students in *n* = 1060 CT scans and reviewed by a team of *n* = 15 radiologists (2–23 years of experience). For *n* = 905, more than two readers segmented each CT scan, after which both segmentation masks were averaged and rounded. Segmentations were performed using Veye Annotator (Aidence BV).

External validation: The external test dataset (*n* = 400) was classified by two readers using CO-RADS. Each case was read twice: first by a radiology resident (F.G., fourth year of training) or radiologist (L.T., 5 years of experience), and thereafter by a certified thoracic radiologist (A.B., 8 years of experience or R.W., 6 years of experience). In cases of discordance or uncertainty, a consensus reading was performed by a third radiologist (A.B., R.W. or L.T.). The distribution of CO-RADS scores for the external test dataset is shown in Table 3.

Segmentations of total lung volumes were performed by a technical physician (K.G.L.) and reviewed by a radiologist (L.T.). In addition, manual segmentations of infectious lung opacities were performed by a radiology resident or radiologist (F.G., L.T.), and reviewed by a certified thoracic radiologist (A.B., R.W.). Segmentations were performed using RVAI (Robovision BV).

### Data preprocessing

To prepare the pixel data from the DICOM series as input for the AI model, quintic interpolation was performed on all slices, yielding a voxel spacing of 1.25 mm × 0.5 mm × 0.5 mm. Subsequently, voxel values were scaled such that the “lung window”, i.e., −1000 to 300 HU, corresponded to the range of −1.0 to 1.0, for numeric stability. Axial slices were extracted from the generated volume and scaled to a fixed size of 256 × 256 pixels.

### Design of the artificial intelligence system

The AI system was designed to delineate COVID-19 infected areas and yield a CO-RADS score through two separate AI models that function in synchrony. First, a convolutional neural network (CNN) with ResUNet-a architecture [13] takes the CT as input and returns two segmentation masks, labelling every voxel in the CT scan as infectious/non-infectious and lung/no-lung. The ResUNet-a architecture for segmentation contained several adjustments (see Supplementary information).

Subsequently, a tree-based ensemble model was used to predict the CO-RADS score. The input features were constructed based on the segmentation masks of the CNN and the corresponding CT image voxel values. The tree-based ensemble model was constructed through a random forest classifier (RandomForestClassifier, scikit-learn v.0.24.1), with the following settings: n_estimators = 300, max_depth = 48, min_samples_split = 12, max_features = 32, and random oversampling with “no majority” strategy (RandomOverSampler, imblearn v0.8.1). All other parameters were at default.

### Statistical analysis

The performance of the AI model’s CO-RADS predictions was evaluated through the weighted Cohen’s kappa score (Eq. 1) since it considers how far the prediction is off.

1 $$ \upkappa =1=\frac{\sum_{i=1}^n{\sum}_{j=1}^n{w}_{ij}{x}_{ij}}{\sum_{i=1}^n{\sum}_{j=1}^n{w}_{ij}{m}_{ij}} $$

with *w* the confusion matrix weights (Supplementary Table S2 for linear), *x* the observed confusion matrix values, *m* the expected confusion matrix values based on chance agreement, and *n* the number of categories.

We implemented the Dice Similarity Coefficient (DSC) to quantify the overlap between the ground-truth label and the AI segmentation in two ways. First, we calculated the DSC (Eq. 1) based on the true positives (TP), false positives (FP), and false negatives (FN) on each individual CT scan. Here, we reported the median DSC and its 95% confidence interval (CI). However, since the negative RT-PCR cases in the test set have no segmented volume, the DSC is not defined (dividing by 0). Therefore, the DSC was only calculated on CT scans of patients with a positive RT-PCR. Secondly, to include false-positive segmentations returned by the AI model for RT-PCR negative CT scans, we included the “micro Dice Similarity Coefficient” (mDSC) as well. Here, the TP, FP, and FN are multiplied by the voxel size (mm^3^) of the respective CT scan. The resulting values over the CT scans are summed, and the mDSC is calculated via Eq. 2. This method yields one value, where larger segmented volumes will have an increased impact on the total score. To analyze the correlation between segmented volumes, we implemented Spearman’s correlation. For statistical tests, *p* < 0.05 was considered significant. See supplemental material for *p* value calculation.

2 $$ DSC=\left(2\ast TP\right)/\left(2\ast TP+ FP+ FN\right) $$

### Model training and deployment

The resulting dataset was divided into a training (*n* = 971) and an internal test (*n* = 89) set, based on a randomly stratified split. Therefore, the ratios of the different CO-RADS classifications were approximately equal in the two sets.

The segmentation model was trained with randomly sampled slices from the training set CT scans. Scaling, rotation, translation, mirroring, and addition of noise were applied to the slices to augment the training data. Stochastic Gradient Descent was used as the optimizer with a learning rate of 0.1 and Nesterov Momentum of 0.9. DSC was implemented as the loss function. The AI model was developed and trained with Tensorflow (v2.3.2).

The classification model was trained on 805 CT scans with 10-fold cross-validation. To account for class imbalance, random over-sampling of minority CO-RADS classification scores was performed.

To perform external validation, the AI model was deployed within the hospital environment and inference was executed on two NVIDIA Quadro RTX 8000.

## Results

### Imaging data

For the ICOVAI dataset, the CT manufacturers were GE (*n* = 424, 33.0%), Siemens (*n* = 499, 38.9%), Philips (*n* = 323, 25.1%), Toshiba (*n* = 37, 2.9%), and unknown (*n* = 3, 0.2%). More detailed acquisition parameters are listed in Supplementary Table S3. For the external validation dataset, chest CT scans were acquired without intravenous contrast in 74.1% patients (*n* = 278), and with intravenous contrast in 25.9% patients (*n* = 97). Distribution of CT manufacturers was GE in 55.7% cases (*n* = 209), and Siemens in 44.3% cases (*n* = 166). Slice thickness ranged from 1.0 to 3.0 mm (average 1.5 mm).

### Internal test: inter-reader agreement

To report on inter-reader agreement with respect to classification using CO-RADS, all scans with a score of at least two readers were analyzed. This analysis also included scans for which no majority consensus could be found, yielding a total of 1058 CT scans. Between all reader pairs (*n* = 4895 combinations), Cohen’s kappa scores were 0.48 (unweighted), 0.72 (linear weighted), and 0.85 (quadratic weighted).

### Internal test: AI performance

The AI model achieved a COVID-19 segmentation DSC of 0.76 and sensitivity of 0.79. The mean true positive, false positive, and false negative volume of COVID were 228.9 mL, 88.3 mL, and 59.1 mL, respectively. The mean absolute error was 117.1 mL.

For total lung segmentation, the AI model achieved a DSC of 0.97 and sensitivity of 0.97. The mean true positive, false positive, and false negative volume of COVID were 4433.9 mL, 97.0 mL, and 137.1 mL, respectively. The mean absolute error was 147.9 mL.

For CO-RADS classification, the AI model achieved Cohen’s kappa scores of 0.58 (not weighted), 0.78 (linearly weighted), and 0.89 (quadratically weighted). The confusion matrix is shown in Table 4.

**Table 4:** Confusion matrix of CORADS classification on internal test set

		Prediction				
		CO-RADS 1	CO-RADS 2	CO-RADS 3	CO-RADS 4	CO-RADS 5
Ground truth	CO-RADS 1	29	1	0	0	0
	CO-RADS 2	3	5	3	1	0
	CO-RADS 3	4	1	1	0	0
	CO-RADS 4	0	2	0	1	3
	CO-RADS 5	0	0	1	2	15

*CO-RADS* COVID-19 Reporting and Data System

### External test: AI performance

**Fig. 2:**
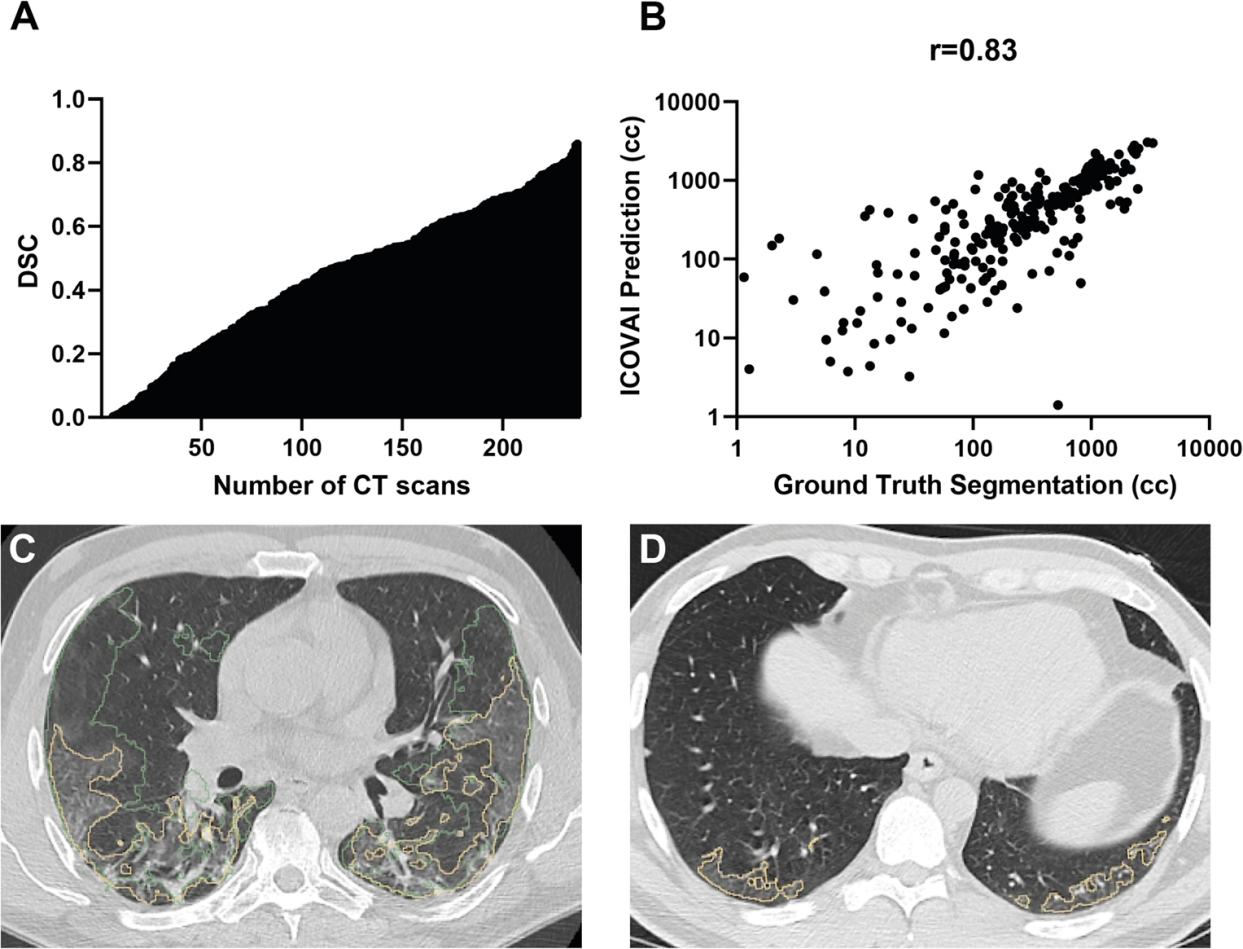
Segmentation of infectious lung opacities by the ICOVAI model on external validation. **A** Distribution of DSC in the external test set of patients with RT-PCR confirmed COVID-19. **B** There is a strong correlation between the volume of infectious lung opacities segmented by the experts (ground truth) and the ICOVAI model. **C** Ground truth segmentations (green contours) included a larger area of discrete ground-glass opacity, versus ICOVAI segmentation (yellow contours) which included only marked ground-glass opacities. **D** False-positive segmentation by the ICOVAI model of normal increased attenuation in the posterior lung bases

The ICOVAI model pipeline excluded *n* = 1 case, leaving *n* = 374 for final analysis. For COVID-19 segmentation, the AI model achieved a performance of 0.59 mDSC and 0.63 sensitivity on the external test dataset, significantly lower than that on the internal test set (*p* < 0.0001). The mean true positive, false positive, and false negative volumes of COVID were 237 mL, 197 mL, and 138 mL, respectively. The mean absolute error was 142 mL (CI: 81–246 mL). The median DSC over all COVID-19–positive CT scans was 0.48. The distribution of DSC scores is shown in Fig. 2A. The correlation between the segmented volume by the AI model and the segmentation by the expert reader was strong (spearman *r* = 0.83, *p* < 0.001); see Fig. 2B.

**Fig. 3:**
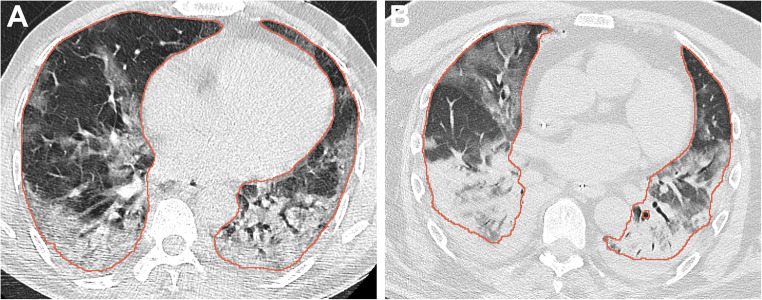
Total lung segmentation (red contours) by the ICOVAI model on the external test dataset was good to excellent, even in cases with extensive ground-glass opacities (**A**). When large consolidations were present, this could lead to false-negative segmentations in the most peripheral parts (**B**)

The total lung segmentation achieved 0.97 mDSC and 0.98 sensitivity on the external test dataset. The mean true positive, false positive, and false negative volumes of COVID were 4.1 L, 178 mL, and 80 mL, respectively. The mean absolute error was 148 mL (CI: 135–156 mL). The median DSC over all COVID-19 positive CT scans was 0.97. Figure 3 shows total lung segmentation in two patients with extensive opacities.

**Fig. 4:**
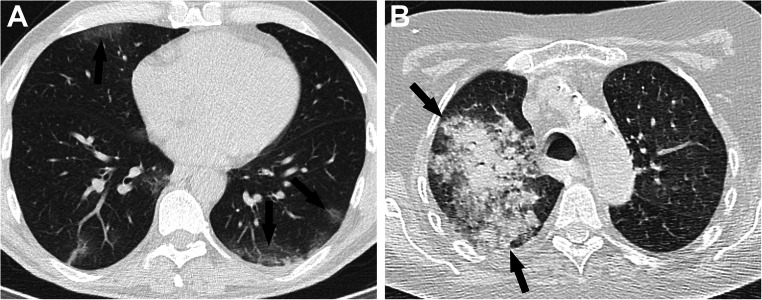
CO-RADS misclassification by the ICOVAI model on the external test dataset. **A** A 55-year-old patient with small subpleural ground-glass opacities in both lungs (arrows), which consisted of a typical appearance of COVID-19 (CO-RADS 5), later confirmed with RT-PCR. The case was misclassified as negative (CO-RADS 1) by the ICOVAI model. **B** A 70-year-old patient was admitted to the intensive care unit with lobar pneumonia, atypical appearance for COVID-19 (CO-RADS 2). CT showed infectious consolidation in the right upper lobe (arrows), and increased attenuation due to hypoventilation in the other pulmonary lobes. The case was misclassified as CO-RADS 5 by the ICOVAI model

The CO-RADS classification achieved Cohen’s kappa scores of 0.41 (not weighted), 0.62 (linearly weighted), and 0.75 (quadratically weighted). See Table 5 for the confusion matrix. Figure 4 shows two examples of misclassification.

**Table 5:** Confusion matrix of CORADS classification on external test set

		Prediction				
		CO-RADS 1	CO-RADS 2	CO-RADS 3	CO-RADS 4	CO-RADS 5
Ground Truth	CO-RADS 1	94	29	9	3	2
	CO-RADS 2	17	35	5	8	3
	CO-RADS 3	12	9	2	3	22
	CO-RADS 4	0	1	4	2	6
	CO-RADS 5	4	6	5	15	78

*CO-RADS* COVID-19 Reporting and Data System

### External test: visual interpretation

A radiologist (L.T., 5 years of experience) performed a qualitative visual inspection of the segmentation results on the external test set. The AI delineation of infectious lung opacities was determined adequate to excellent for the majority of cases. When compared to the ground truth labels generated by the radiologists, the ICOVAI model was less sensitive to discrete ground-glass opacities. In several cases, the ICOVAI model generated false-positive segmentations of non-infectious lung opacities such as atelectasis or fibrosis.

## Discussion

In this multicenter study, we described the development of the ICOVAI model and performed an independent external validation using data from five institutions. We observed a significant reduction in performance on the external test as compared to the internal test for lung opacity segmentation and CO-RADS prediction, but not for lung contour segmentation.

To the best of our knowledge, we have performed the first pre-market external validation study that independently assessed the segmentation performance of a COVID-19 imaging AI solution using large-volume multicenter data. Our work shows that published results of COVID-19 segmentation on internal test sets may not generalize well to patient data from other institutions.

External validation can highlight the shortcomings of a predictive model, which were not apparent during internal testing on CT scans sampled out of the same cohort. The importance of external validation is illustrated by the increasing number of high-impact journals requesting it for all predictive models before publication [14]. Moreover, repetitive test set use by slightly different experiments can lead to “test set overfitting” [15], where the model fits the test data well by chance in one of the experiments. We solved these problems with external validation, where the model is tested once at an external location with an unrelated dataset.

The reported differences in segmentation performance of COVID-19 pneumonia on the internal versus external datasets may partially be explained by interreader variation. The lung areas labelled as abnormal by annotators of the development dataset versus independent annotators of the external dataset may vary because of variations in default window-level settings on the different annotation platforms used to perform the ground truth segmentations, leading to distinct cut-off values to label lung densities (Fig. 2C).

Variation in CO-RADS scoring between the internal and the external test sets could, to some extent, be explained by selection bias. For the internal dataset, CO-RADS scores were excluded when there was no majority consensus between readers, eliminating the “hardest to evaluate” cases. This is most likely also the cause for the AI model’s kappa score being higher than the inter-reader kappa score. When results on the internal test set are better than the ground truth, test set overfitting may be occurring [15]. In this case, external validation can reflect the true, tempered performance of the AI model more accurately.

A prior study by Lessmann et al trained an AI system with single-center data to score the likelihood of COVID-19 using CO-RADS [16]. They found a moderate to substantial agreement between observers, reporting a linearly weighted kappa of 0.60 on their internal test set, and 0.69 on their external test set. In our multicenter study, we found a similar level of agreement (kappa values of 0.78 and 0.62, respectively). Previous multicenter studies that included external validation have focused on a binary or ternary classification of COVID-19 versus other types of pneumonia and normal lungs [17–22]. These studies reported a high to outstanding area under the receiver operating curve (AUC) (0.87–0.98) for identifying COVID-19 on CT. However, the results are difficult to compare with our study that focused on predicting CO-RADS, a more complex multicategorical assessment scheme. Additionally, our external validation was executed by independent researchers. Similarly, Jungmann et al performed an independent external validation on four commercial AI solutions to differentiate COVID-19 pneumonia from other lung conditions [23]. They found high negative predictive values (82–99%) for the tested models, but deemed only one solution to have an acceptable sensitivity. The specificity of the four solutions was highly variable (31–80%) and positive predictive values were low (19–25%). Their study was limited to evaluating binary classification and did not assess the segmentation accuracy. Regarding lung opacities segmentation performance on COVID-19 patients, the multicenter study of Zhang et al reached an mDSC of 0.55–0.58 on internal testing, comparable with our findings on external validation [20]. Other published studies have reported higher DSC values for segmentation of lung opacities. However, most studies used single-center data, datasets of limited size, or did not perform external validation [24–27].

Our study has several limitations. First, patients were selected by convenience sampling, which may have introduced selection bias. The internal dataset included controls from the National Lung Screening Trial that did not correspond to the target population. This was mitigated by performing an independent validation with a balanced external dataset. Secondly, CO-RADS is prone to interobserver variability and is therefore an imperfect reference standard. Cases in the internal dataset were excluded when there was a disagreement between all readers on CO-RADS classification, arguably inducing a bias towards less complicated cases. For the external dataset, disagreements were resolved using consensus. Thirdly, interobserver variability of COVID-19 segmentations was not evaluated. Therefore, we cannot determine whether the ICOVAI model was reasonably close to the agreement between radiologists.

Future AI developers might benefit from a centralized, high-quality reference image repository to perform external validation of their model, which would also be helpful in setting benchmarks of model performance.

## Conclusion

This study evaluated the ICOVAI model performance independently using an external, multicenter test dataset. Segmentation of total lung volumes in both internal and external datasets was excellent, even in patients with severe COVID-19 pneumonia. The performance of the ICOVAI model on segmentation of infectious lung opacities and classification of CO-RADS was significantly worse on the external test dataset compared to the internal test dataset. The results showed limitations in the generalizability of the ICOVAI model, therefore restricting the potential for clinical use. Our study demonstrates the importance of independent external validation of AI models.

## Supplementary Information

ESM 1 (DOCX 22.9 kb)

## Declarations

### Guarantor

The scientific guarantor of this publication is Kevin Groot Lipman.

### Statistics and biometry

No complex statistical methods were necessary for this paper.

### Methodology

• retrospective

• diagnostic study

• multicenter study

## Abbreviations

AI: Artificial intelligence
CNN: Convolutional neural network
CO-RADS: COVID-19 Reporting and Data System
COVID-19: Coronavirus disease 2019
DICOM: Digital Imaging and Communications in Medicine
DSC: Dice Similarity Coefficient
ICOVAI: International Consortium for COVID-19 Imaging AI
RT-PCR: Reverse transcriptase-polymerase chain reaction
