# Supplementary material for: Is the generalizability of a developed artificial intelligence algorithm for COVID-19 on chest CT sufficient for clinical use? Results from the International Consortium for COVID-19 Imaging AI (ICOVAI)
Source: Eur Radiol. 2023 Jan 18;33(6):4249–58. doi: 10.1007/s00330-022-09303-3 (PMC9848031; doi:10.1007/s00330-022-09303-3)
Supplement: Supplementary file 1 — (DOCX 22.9 kb) [file 330_2022_9303_MOESM1_ESM.docx]

**Supplemental Material**

**ResU-Net-a architecture changes**

Adjustments of ResU-Net-a architecture: (1) The PSP pooling layers were omitted, (2) five stages were implemented instead of six, (3) sixteen filters were used in all five stages instead of exponentially increasing the filters, (4) the dilations per stage in the ResNets were adjusted (Supplementary Table S1), (5) Instance Normalization [28] was implemented instead of Batch Normalization, and (6) transpose convolutions were used to upsample.

**Calculation of p-values**

Significant differences were calculated through bootstrapping, since both the kappa scores and de mDSC are a single value for the entire dataset. The external dataset was bootstrapped 10000 times with replacement, yielding mean and standard deviation of the returned kappa and mDSC scores. Subsequently, the z score from this distribution was calculated by Equation 3. The scipy package (v.1.2.3, stats.norm.sf) was used to convert the z-score to the corresponding p-value (two-tailed).

$z = (x-\mu)/ (\sigma)$ (Eq 3)

with *z* the z-score, x the performance measure on the internal test set (kappa/mDSC), $\mu$ the mean and $\sigma$ the standard deviation of the bootstrapped distribution of external test set’s performance measures.

**Table S1. Adjustments to the original Res-Unet architecture. Dilations are the ‘field of view’ over which the upsampling is performed.**

| Stage | Dilation |
| --- | --- |
| 1 | (1, 9, 17) |
| 2 | (1, 5, 9) |
| 3 | (1, 3, 5) |
| 4 | (1, 2, 3) |
| 5 | (1) |

**Table S2. The linear weights used to calculate the Cohen’s kappa for the CORADS classification task. The larger the difference between the CO-RADS score of the radiologist and the AI prediction, the higher the penalty.**

| **CO-RADS** | | **ICOVAI model** | | | | |
| --- | --- | --- | --- | --- | --- | --- |
|  |  | **1** | **2** | **3** | **4** | **5** |
| **Radiologists** | **1** | 0 | 1 | 2 | 3 | 4 |
|  | **2** | 1 | 0 | 1 | 2 | 3 |
|  | **3** | 2 | 1 | 0 | 1 | 2 |
|  | **4** | 3 | 2 | 1 | 0 | 1 |
|  | **5** | 4 | 3 | 2 | 1 | 0 |

AI: Artificial Intelligence; CO-RADS: COVID-19 Reporting and Data System; ICOVAI: International Consortium for COVID-19 Imaging AI

**Table S3. CT scan acquisition parameters of the ICOVAI dataset, denoted per task and specific set.**

|  | | **Classification** | | **Segmentation** | |
| --- | --- | --- | --- | --- | --- |
|  |  | **Training** | **Internal test** | **Training** | **Internal test** |
| **Slice Thickness** | **Min** | 0.5 | 0.625 | 0.5 | 0.625 |
|  | **Median** | 1.0 | 1.0 | 1.0 | 1.0 |
|  | **Max** | 3.2 | 3.2 | 5.0 | 3.2 |
|  | **Mean** | 1.202 | 1.213 | 1.188 | 1.156 |
|  | **Count < 2 mm** | 657 | 57 | 800 | 74 |
|  | **Count >= 2 mm** | 148 | 15 | 171 | 15 |
|  | **Total** | 805 | 72 | 971 | 89 |
| **Slice Spacing** | **Min** | 0.45 | 0.45 | 0.45 | 0.45 |
|  | **Median** | 1.0 | 1.0 | 1.0 | 1.0 |
|  | **Max** | 3.0 | 3.0 | 3.0 | 3.0 |
|  | **Mean** | 1.089 | 1.052 | 1.076 | 1.019 |
|  | **Total** | 805 | 72 | 971 | 89 |
| **X-ray tube current** | **Min** | 40.0 | 50.0 | 40.0 | 50.0 |
|  | **Median** | 100.0 | 90.0 | 100.0 | 85.0 |
|  | **Max** | 499.0 | 160.0 | 499.0 | 160.0 |
|  | **Mean** | 135.2 | 98.6 | 132.3 | 90.7 |
|  | **Total** | 160 | 14 | 179 | 14 |
| **Kilo voltage peak** | **Min** | 80.0 | 80.0 | 80.0 | 80.0 |
|  | **Median** | 120.0 | 110.0 | 120.0 | 100.0 |
|  | **Max** | 140.0 | 140.0 | 140.0 | 140.0 |
|  | **Mean** | 111.5 | 110.1 | 111.063 | 109.1 |
|  | **Total** | 804 | 72 | 969 | 89 |

ICOVAI: International Consortium for COVID-19 Imaging AI

**References**

28. Ulyanov D, Vedaldi A, Lempitsky V (2016) Instance normalization: the missing ingredient for fast stylization. arXiv:1607.08022 [cs.CV] 10.48550/arXiv.1607.08022
